# Cardiac Tumor Within the Left Ventricle: Thrombus or Something Else?

**DOI:** 10.1016/j.jaccas.2024.102719

**Published:** 2024-12-04

**Authors:** Kyle T. Barron, Archie Landrum, Hussein Abu Daya, Stephen Clarkson

**Affiliations:** University of Alabama Birmingham, Birmingham, Alabama, USA

**Keywords:** echocardiography, imaging, multidisciplinary, multimodality, tumor

## Abstract

Papillary fibroelastoma is a benign cardiac tumor that most commonly arises from the valvular endocardium and is typically resected because of its predisposition to cause embolic complications. In this clinical vignette, we describe the case of a patient who presented with a round, mobile 1.3 × 1.1-cm sessile mass attached to the left ventricular apex discovered on transthoracic echocardiography at an outside institution. A multidisciplinary team and multimodality imaging approach were taken to treat a diagnostically challenging presentation of a papillary fibroelastoma due to lack of visualization on higher-resolution imaging studies.

## Graphical Abstract

**Figure:**
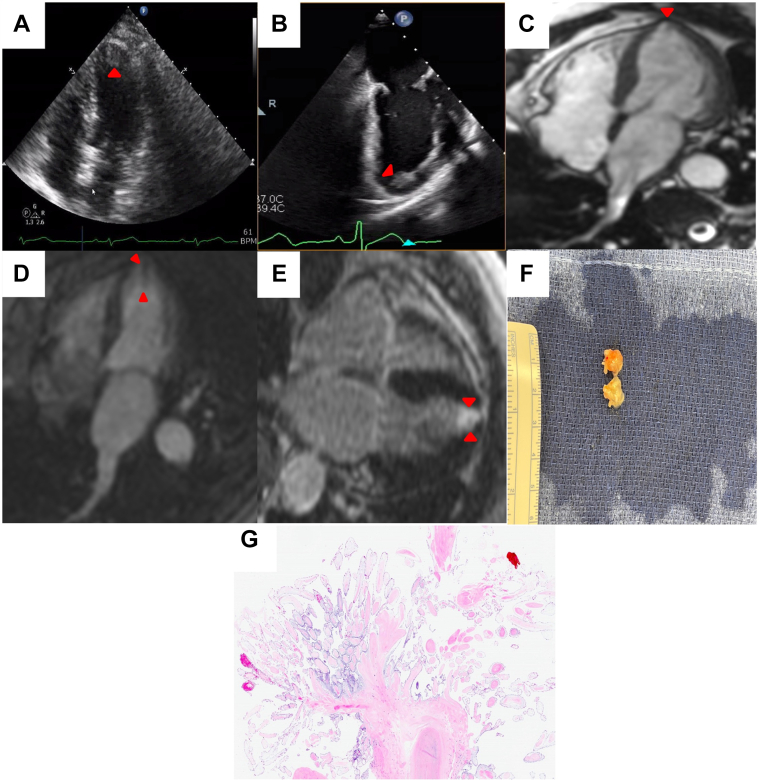

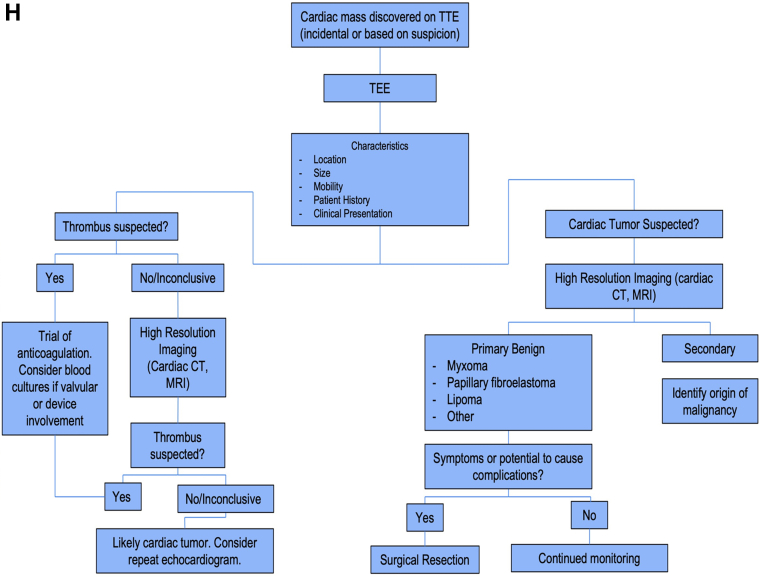

## Case Summary

**Figure 1:**
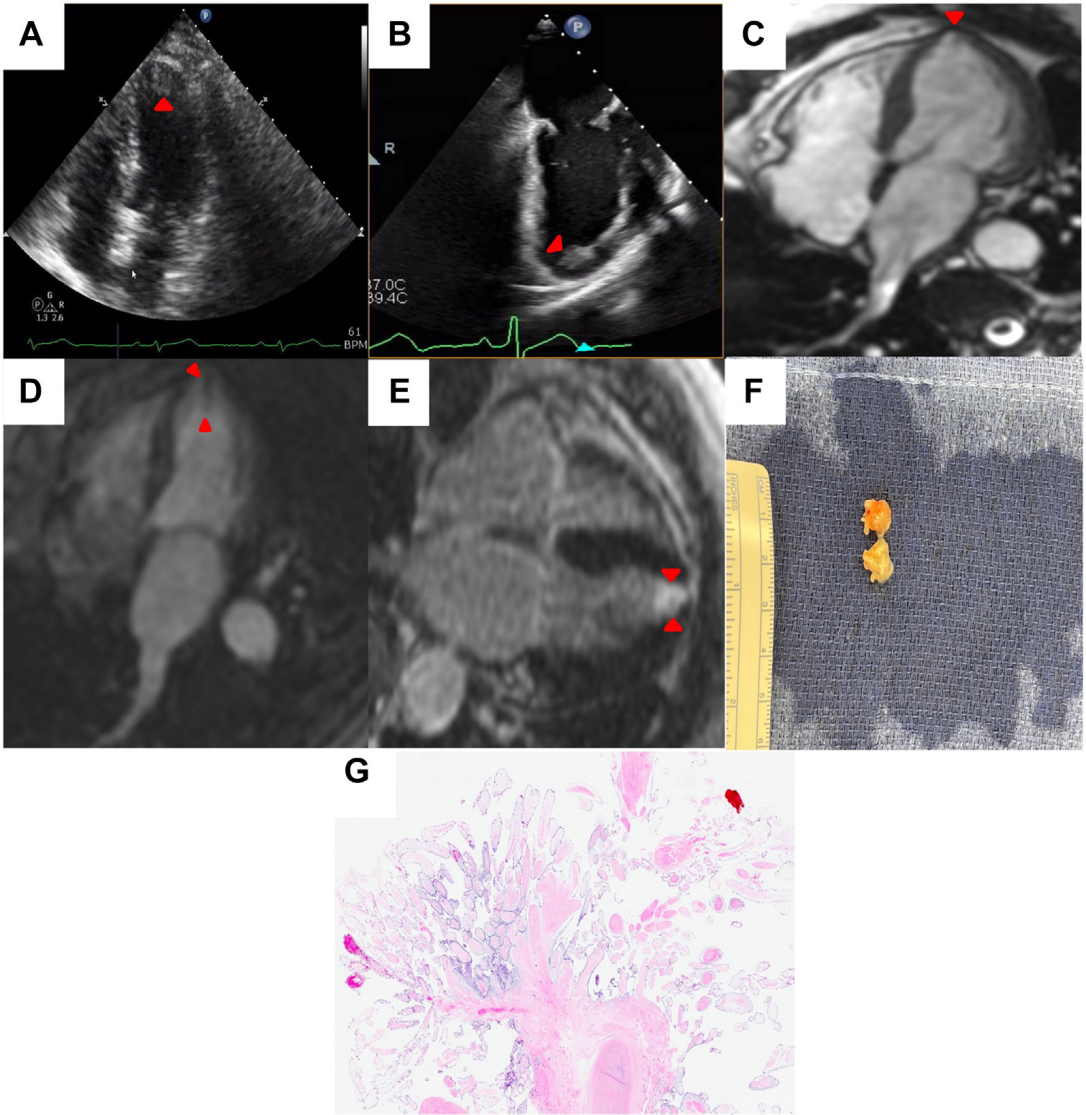

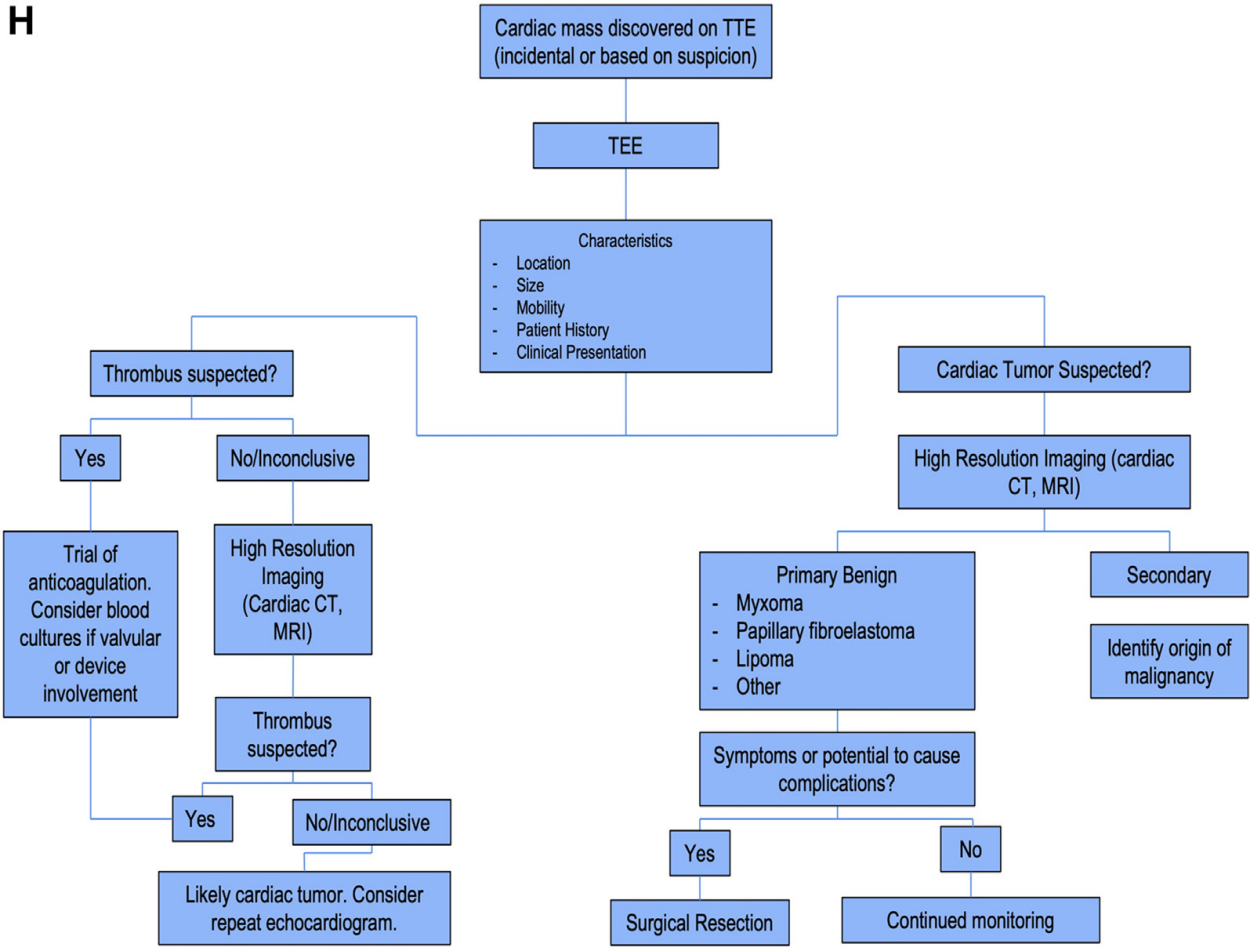
Left Ventricular Papillary Fibroelastoma (A) Transthoracic echocardiogram apical 4-chamber view demonstrating mobile mass in the left ventricular apex (arrowhead). (B) TEE mid-esophageal long-axis view better visualizing the left ventricular apical mass (arrowhead). (C) Cardiovascular magnetic resonance at 1.5-T showing 4-chamber steady-state view demonstrating apical wall motion abnormality (arrowhead). (D) Steady-state free precession cine imaging displaying 5-mm linear filling defect at the left ventricular apex following intravenous infusion of gadolinium contrast (arrowheads). (E) Four-chamber view demonstrating enhancement of the left ventricular apex 10 minutes after intravenous infusion of gadolinium contrast indicating fibrotic structure (arrowheads). (F) Image of the surgically resected left ventricular apical mass. (G) Histopathologic imaging using hematoxylin-eosin staining demonstrating characteristic appearance of central stalk composed of collagen and elastin with frond-like projections confirming the diagnosis of the mass as a papillary fibroelastoma. (H) Flowchart depicting approach to workup and management of cardiac masses. CT = computed tomography; MRI = magnetic resonance imaging; TEE = transesophageal echocardiogram; TTE = transthoracic echocardiogram.

A 67-year-old woman presented to her local hospital with complaints of shortness of breath and palpitations. Her medical history was notable for hypertension, hyperlipidemia, hypothyroidism, and stage 1 invasive lobular carcinoma of the breast that was successfully treated with lumpectomy, chemotherapy, and radiation. Her vitals and physical exam on admission were unremarkable. In her initial workup, transthoracic echocardiogram demonstrated a round, 1.3 × 1.1 cm in size, irregular, sessile, mobile mass attached to the left ventricular apical myocardium with no wall motion abnormalities (Figure 1A, Video 1) that was confirmed on transesophageal echocardiogram (TEE) (Figure 1B, Videos 2 and 3). The patient was prescribed apixaban (5 mg twice daily) and referred to our institution for surgical evaluation.

**Take-Home Messages**

• The patient’s clinical presentation and history as well as different diagnostic characteristics of cardiac masses should be taken into consideration to guide evaluation when using a multimodal imaging approach.
• Timely management of cardiac tumors dependent on their characteristics and location can help prevent adverse patient outcomes.

Electrocardiogram obtained on transfer demonstrated normal sinus rhythm with occasional premature supraventricular contractions. A dedicated cardiac computed tomography was unable to demonstrate the mass. Cardiac magnetic resonance imaging (MRI) with gadolinium contrast was unable to visualize the mass and instead showed a suspected regional wall motion abnormality on 4-chamber steady-state cine sequence (Figure 1C, Video 4). There was a 5-mm thin, linear structure with signal appearance similar to myocardium with small filling defect on first pass perfusion (Figure 1D) and an area of high signal intensity late after infusion in the left ventricular apical wall (Figure 1E). This combination of findings was speculated to be the result of a prior small apical infarction that caused a corresponding wall motion abnormality with development of a thrombus that had resolved with anticoagulation. A repeat TEE was performed due to conflicting data that demonstrated the same apical mass seen on prior echocardiograms. Following surgical resection of the mass, gross examination demonstrated a papillary fibroelastoma (Figure 1F) confirmed on pathological analysis (Figure 1G). Her apixaban (5 mg twice daily) was continued and subsequent transthoracic echocardiograms at 3 and 4 months postoperative did not display any remnants of the removed mass.

The leading differential diagnosis based on initial echocardiography studies was myxoma or papillary fibroelastoma owing to the lack of risk factors for development of left ventricular apical thrombus that includes normal ejection fraction, no wall motion abnormalities, no history of coronary artery disease or recent myocardial infarction, and no aneurysm. The findings on MRI presented a difficult diagnostic challenge owing to lack of visualization of the mass after sustained treatment with apixaban (5 mg twice daily). This clinical scenario could reasonably lead to the conclusion that the mass was a thrombus, especially given that cardiac MRI is often considered the gold standard for assessing cardiac tumor characterization, localization, and tissue invasiveness when compared with echocardiography.^1^ These findings and the similar echocardiographic characteristics shared among thrombi, myxomas, and papillary fibroelastomas including their high degree of mobility and echo-density when compared with myocardium led to the need for repeat TEE, which should be used in cases when high-resolution studies fail to visualize the mass and suspicion for cardiac tumor remains high (Figure 1H).^2^^,^^3^ Ultimately, utilization of an interdisciplinary heart team approach was crucial in this complex case of papillary fibroelastoma, which was resected before development of systemic embolic complications.

## Funding Support and Author Disclosures

The authors have reported that they have no relationships relevant to the contents of this paper to disclose.

## Appendix

Video 1 Transthoracic Echocardiography Apical 4-chamber view demonstrating mobile mass in the left ventricular apex.

Video 2 Transesophageal Echocardiography Mid-esophageal long-axis view better demonstrating and defining papillary fibroelastoma within the left ventricular apex.

Video 3 Transesophageal Echocardiography Zoomed in mid-esophageal long-axis view of the left ventricular apical papillary fibroelastoma for enhanced visualization and characterization.

Video 4 Cardiac Magnetic Resonance 4-Chamber Steady-State Cine Sequence Demonstration of small apical wall motion abnormality.
